# The Seventeenth International Congress of Medicine

**Published:** 1913-07-26

**Authors:** 


### THE SEVENTEENTH INTERNATIONAL CONGRESS OF MEDICINE.

### Notabilities and Notable Items.

The whole of the very extensive arrangements for the sessions of the forthcoming International Congress in London are practically settled; and the complicated organisation necessary has been most skilfully created and set in motion. The sixth circular, in which the programme is set forth, has been published, and runs to fifty-eight large pages. This means, of course, that a prodigious number of papers are to be read and discussed, and must have entailed unceasing work for the executive committee and the secretariat. A glance through the list of those who are down to read papers before the various sections is sufficient to reveal the extraordinary authority with which the subjects will be discussed. Of the leaders of scientific medicine, on both sides of the Atlantic, a very large percentage seem to be coming to London to discourse each on the problem he has done most to elucidate. Seldom, if ever, can so representative a gathering of the flower of the medical profession have taken place as that which promises to be seen in London during the second week of August.

Prince Arthur of Connaught has consented to open the Congress on behalf of the King. The Opening Meeting will take place in the Albert Hall at 11 A.M. on Wednesday, August 6; and the Closing Meeting at 4 P.M. on Tuesday, August 12. The President of the Congress is Sir Thomas Barlow, and the General Secretary Dr. W. P. Herringham. On each day of the Congress, except Saturday, there will be a General Session, as well as the Sectional Sessions; and at each General Session a General Address will be delivered. On August 6 is the Address on Medicine by Professor Chauffard, of Paris; on August 7 that on Surgery by Professor Harvey Cushing, of Harvard; on August 8 on Pathology by Professor Ehrlich, of Frankfort; on August 11 on Heredity by Professor Bateson; and on August 12 on Public Health by Mr. John Burns, M.P.

Space prevents any full account of the sectional programmes, which are packed full of the most interesting matters. Some of the papers and discussions look as if they might be of exceptional interest. Thus, two Toronto workers, Dr. Collip and Professor Macallum, are reading a paper on a Hitherto Unrecognised Substance in Nerve Cells. In the Section of General Pathology a discussion on Shock is to be opened by Dr. Crile and Professor Henderson, the two Americans whose antagonistic theories as to the causation of this condition have been so much debated this last few years. This battle of the giants should be a most exciting affair. In Bacteriology Professor von Gruber's paper on the Nature of Virulence sounds especially attractive.

In the Section of Therapeutics English-speaking authors are vastly outnumbered by those who write in French or German. Professor Cushing's Report on the Action and Use of Remedies for Pain and Sleeplessness raises an old topic, and may shed more light on its obscurities. Medicine, as one might expect, commands many interesting papers. Professor Brill is to describe the disease now usually known by his name: the title of his paper conveys the impression that he is not quite sure even now whether this is or is not a mild form of typhus fever. Several papers on aspects of the tuberculosis problem are set down; as also on diabetes, of which one optimistic Portuguese member of the Congress describes a radical cure. Experimental Cirrhosis of the Liver is another paper in this section which contains possibilities.

In the Section of Surgery Professor Matas and Professor von Oppel report on the Surgery of the Arterial System; the former has attained the distinction of having had his name attached to an operation for aneurysm, which a few years ago was regarded as impossible of performance. Another eponymous innovation is the celebrated "Fowler posture" after operations, which will be described by Dr. Fowler himself. The Influence or Radium on Malignant Growths is the thesis of Drs. Wickham and Degrais, of Paris, two very well-known workers in this field of research. In Orthopædics there is very little British talent to the fore, but any amount of Continental and American papers are to be read: we have remarked before on the comparative neglect of this subject in Britain. All the leading innovators in anæsthetics seem to be coming to the Congress; Ferguson (open ether), Burkhardt (intravenous ether), Cunningham (rectal administration), Tuffier (spinal anæsthesia), Teter (nitrous oxide with oxygen, for major surgery), and others. In this subsection, too, a preliminary skirmish of the Crile-Henderson controversy is apparently to take place, a sort of reconnaissance for the big affair of two days later.

In Gynæcology there is little that appears likely to be epoch-making. Wertheim will discourse on Uterine Cancer; and most of the other topics for discussion are along stereotyped lines. In Ophthalmology many well-known and original surgeons will read papers. Colonel Elliot on Glaucoma and Colonel Smith on Cataract are bound to be instructive; and Dr. Maitland Ramsay's colour photographs of eye diseases will doubtless prove an attraction. The Section of Diseases of Children is not overloaded with papers: a novelty is promised by Dr. Weiss in the shape of a New Diagnostic Symptom of Scurvy Rickets. The Neurological Section, on the other hand, has a very big list to get through: many leaders of this specialty whose names are household words are to be found on it.

In the Section of Dermatology and Syphilis, too, many English and foreign experts of recognised eminence are to read papers. Perhaps Ehrlich on Salvarsan is the *pièce de résistance* in this section. In Urology there is nothing of great moment, as far as one can judge from the titles of communications. In Rhinology and Laryngology a great number of papers are put down, most of them by Continental surgeons; and the same is true of Otology, which is allotted a separate section. In the Section of Hygiene appears the first paper of great institutional importance—namely, Dr. McWalter's Plea for State-Endowed Hospitals and a State Medical Service. The remaining sections have all questions of interest to discuss; that of the History of Medicine has the heaviest programme: no less than sixty-nine papers have been promised, many of them of great interest.

